# Rapid chronic kidney disease progression in younger, First Nations patients in the Northern Territory

**DOI:** 10.1111/imj.70062

**Published:** 2025-04-22

**Authors:** Winnie Chen, Oyelola Adegboye, Gillian Gorham, Asanga Abeyaratne, Sandawana William Majoni, Sean Taylor, Samuel Heard, Harshana Munasinghe, Matthew J. L. Hare, Alan Cass

**Affiliations:** ^1^ Menzies School of Health Research Charles Darwin University Darwin Northern Territory Australia; ^2^ Department of Nephrology Royal Darwin Hospital, NT Health Darwin Northern Territory Australia; ^3^ School of Population and Global Health University of Melbourne Melbourne Victoria Australia; ^4^ Central Australian Aboriginal Congress Alice Springs Northern Territory Australia; ^5^ Endocrinology Department Royal Darwin Hospital, NT Health Darwin Northern Territory Australia

**Keywords:** Indigenous health, chronic kidney disease, rural health services, kidney replacement therapy, dialysis

## Abstract

This 6‐year retrospective cohort study investigated the progression of chronic kidney disease (CKD) among Australian First Nations patients in remote Northern Territory. We found that younger patients (age less than 50 years) were at a higher risk of progression to kidney replacement therapy or death compared to older adults. The results indicate a need to target early CKD intervention efforts to younger patients at risk of rapid progression.

Remote Northern Territory (NT) communities in Australia are a hotspot for chronic kidney disease (CKD).^1^ Australian First Nations people in the NT have over 30% prevalence of CKD compared to approximately 10% in non‐First Nations people, with a much higher prevalence of end‐stage kidney disease (ESKD).^2^, ^3^ As with other First Nations populations globally, the heavy burden of CKD in remote NT is associated with social disadvantage.^4^ The Australian and New Zealand Dialysis and Transplant (ANZDATA) registry data reports the median age of dialysis initiation in the NT to be 51 years – more than a decade younger than the median age (63 years) across all dialysis centres in Australia and New Zealand.^5^ Despite recognition that First Nations patients commencing dialysis are younger, there remains limited understanding regarding differences in CKD progression by age. In this study, we aimed to examine the differences in CKD progression by age among First Nations people in the remote NT.

This was a retrospective cohort study using data from the Territory Kidney Care database, covering a 6‐year follow‐up period from 2017 to 2023, with longitudinal data available in 3‐monthly intervals. The database contains individual‐level electronic health record (EHR) data from all NT public hospitals (*n* = 6), all NT Health remote primary health clinics (*n* = 56) and the majority of Aboriginal Community Controlled Health Organisations (ACCHOs) in the NT (*n* = 11).^6^, ^7^ In remote NT, NT Health and ACCHO clinics are the sole primary healthcare providers, serving predominantly First Nations patients. Clinical information extracted included demographic details, coded diagnoses, and laboratory results. The presence of CKD and related chronic conditions is determined using previously validated EHR‐based algorithms.^8^ The inclusion criteria for this study were First Nations patients currently attending a participating remote primary health clinic, older than 16 years of age, with CKD stage 3a to CKD stage 5. CKD patients already on kidney replacement therapy (KRT) at baseline were excluded from the study.

Medians and interquartile ranges (IQRs) were reported for continuous variables, while categorical variables were presented as counts with percentages. The primary outcome of interest was time to a combined endpoint of KRT or death, with a focus on describing differences in outcomes between those who are younger (<50 years) and older (≥50 years). This age cut‐off was based on the median age of dialysis initiation in the NT (51 years).^5^ Secondary outcomes included time to KRT and time to death. Unadjusted and adjusted Cox proportional hazards (PH) were used to describe the association between age categories and the primary outcome. As KRT and death are competing risks, an additional competing risk analysis was performed. Cumulative incidence function for KRT and death according to age category, and results of the Fine‐Gray model were reported. Statistical analyses were conducted in R (version 4.2.3)^9^ and Python (version 3.9.12).^10^ Ethics approval was granted by NT Health and Menzies School of Health Research Human Research Ethics Committee (NTHREC 2021‐4102) and was approved by the NT Health Research Governance Office.

The study cohort consisted of 1129 First Nations patients with baseline CKD stages of 3a to 5 (eGFR <60 mL/min per 1.73 m^2^) (Table 1). At baseline, the median age was 60 years, with 36% males. Common comorbidities included hypertension (prevalence 88%), diabetes (78%) and coronary artery disease (37%). Comorbidities were more common in patients with more severe stages of CKD.

**Table 1:** Characteristics of included patients at baseline 2017, by CKD stage

Characteristic	CKD 3a, *n* = 594	CKD 3b, *n* = 282	CKD 4, *n* = 168	CKD 5, *n* = 85	All, *n* = 1129
Age	61 (21)	61 (19)	57 (17)	57 (17)	60 (19)
Gender (M)	229 (39%)	92 (33%)	56 (33%)	27 (32%)	404 (36%)
Baseline eGFR†	54 (11)	38 (11)	21 (7)	9 (5)	44 (25)
Baseline uACR†	24 (111)	86 (271)	218 (282)	270 (310)	69 (238)
Comorbidities					
Diabetes	433 (73%)	227 (80%)	147 (88%)	79 (93%)	886 (78%)
Hypertension	502 (85%)	252 (89%)	160 (95%)	82 (96%)	996 (88%)
Coronary artery disease	205 (35%)	102 (36%)	69 (41%)	39 (46%)	415 (37%)
Cerebral vascular disease	53 (8.9%)	28 (9.9%)	20 (12%)	4 (4.7%)	105 (9.3%)
Peripheral vascular disease	26 (4.4%)	15 (5.3%)	6 (3.6%)	5 (5.9%)	52 (4.6%)
Obesity	133 (22%)	71 (25%)	42 (25%)	18 (21%)	264 (23%)
Rheumatic heart disease	87 (15%)	35 (12%)	22 (13%)	13 (15%)	157 (14%)

Median (IQR) for continuous variables; *n* (%) for categorical variables. Percentages rounded to two significant figures.

† Missing values are excluded for eGFR (*n* = 25 individuals) and uACR (*n* = 186 individuals).

CKD, chronic kidney disease; eGFR, estimated glomerular filtration rate; uACR, urine albumin creatinine ratio.

Figure 1 presents Kaplan–Meier survival curves of time to KRT or death, comparing younger (<50 years) versus older (≥50 years) age categories. The Kaplan–Meier curves show that median survival to the combined endpoint of KRT or death for younger patients was approximately 3.6 years, compared with 5.6 years in older patients. At the end of the 6‐year follow‐up period, a higher proportion of younger patients progressed to KRT or death (65%) compared to the older group (43%) – see also Table S2. In the unadjusted Cox PH model, younger patients <50 years were at higher risk of progressing to KRT or death compared to older patients ≥50 years (unadjusted HR 1.52, 95% CI 1.21–1.92). When adjusted for gender and comorbidities (diabetes, cardiovascular disease, hypertension), the effect of age category on risk of progression to KRT or death was strengthened (adjusted HR 2.17, 95% CI 1.71–2.75) (Table 2). Figure 2 presents cumulative incidence functions for competing risks of KRT and death by age category. Fine‐Gray's test showed that, when accounting for these two competing risks, the cumulative incidence of KRT remains significantly higher in the younger age category. Full results are available in Appendix S1.

**Figure 1:**
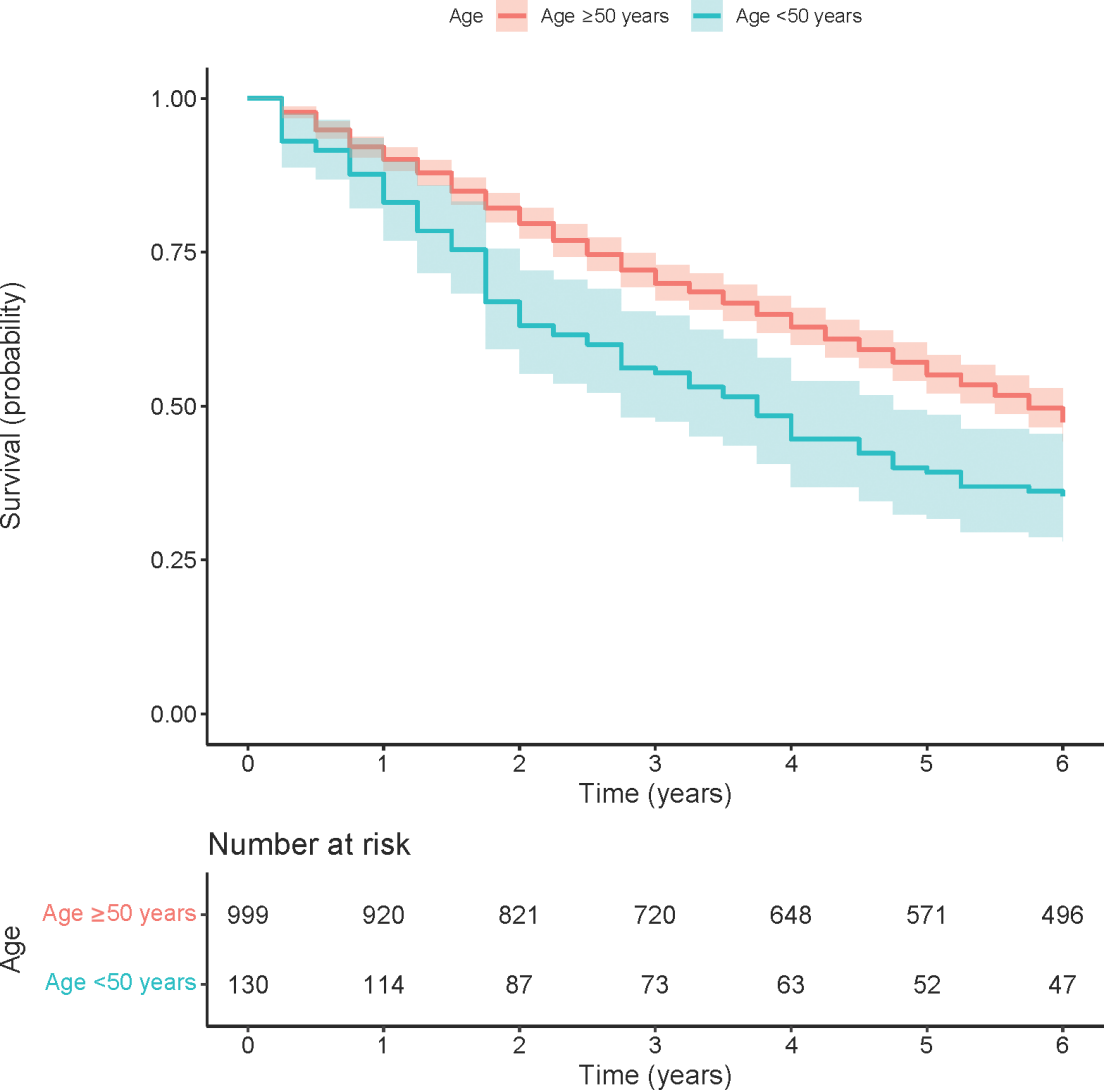
Kaplan–Meier survival curves for time to combined outcome of kidney replacement therapy or death, by age category. Red represents individuals age ≥50 years, and blue represents individuals <50 years. The number at risk at each time interval, in years, is shown below the graphs.

**Table 2:** Cox proportional hazard model results, by age category

Outcome	Variable	HR	95% CI lower	95% CI upper	*P*‐value
Combined outcome of KRT or death unadjusted	Age < 50	1.52	1.21	1.92	<0.001
Combined outcome of KRT or death adjusted†		2.17	1.71	2.75	<0.001
KRT unadjusted	Age < 50	3.12	2.34	4.15	<0.001
KRT adjusted†		4.92	3.66	6.62	<0.001
Death unadjusted	Age < 50	0.62	0.40	0.95	0.030
Death adjusted†		0.80	0.52	1.24	0.323

† Adjusted Cox proportional hazard includes gender, diabetes, cardiovascular disease and hypertension.

CI, confidence interval; HR, hazard ratio; KRT, kidney replacement therapy.

**Figure 2:**
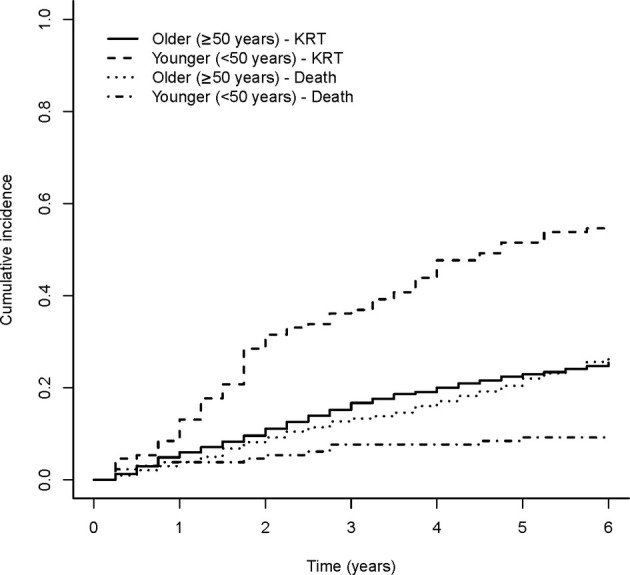
Cumulative incidence functions for competing risks of kidney replacement therapy and death, by age category.

## Discussion

While it is well established that First Nations Australians start KRT earlier than non‐First Nations Australians, our study is a first in investigating age‐related differences in CKD progression among a high‐risk group of First Nations patients. We found that within 4 years, over half of younger First Nations patients (<50 years) with CKD in this NT cohort progressed to KRT or had died. We demonstrated that younger patients were at significantly higher risk of progression to KRT compared to older patients, even when the competing risk of death is taken into account. Additional analyses confirmed that age‐related differences remained present when patients were further stratified by baseline CKD stages.

The accelerated progression highlights an urgent need to optimise early management of CKD and target resources towards this higher‐risk cohort. Preventing rapid progression to KRT requires multifaceted approaches, including addressing upstream factors and underlying inequities within healthcare provision, access to culturally safe, high‐quality care for CKD and related chronic conditions in primary healthcare, timely diagnosis and timely access to specialist‐supported care.^4^

A previous prospective cohort study of First Nations people across northern and central Australia did not show age to be a significant risk factor for CKD progression in terms of eGFR decline or progression to a combined renal endpoint.^11^ However, few patients in that study (*n* = 85, 15.5% of the study cohort) had eGFR <60 mL/min per 1.73 m^2^, whereas all 1129 patients in our cohort were CKD patients with eGFR <60 mL/min per 1.73 m^2^ (inclusion criteria stages 3a or higher). Similar to our study, a population‐wide Tasmanian study of patients with CKD stage 4 and above (eGFR <30 mL/min per 1.73 m^2^) showed the risk of death to increase with age, with the risk of KRT decreasing with advanced age.^12^ However, the Tasmanian study did not access primary care data and was conducted in a much older cohort (median age 81 years, vs median age 60 years in our study).

A strength of this study is that it used linked data from the Territory Kidney Care database, which included longitudinal EHR data from all NT public hospitals and the majority of remote NT Health and ACCHO clinics. This allowed for accurate categorisation of CKD staging, comorbidities and study endpoints (KRT and death). Nevertheless, this was a retrospective study that relied on secondary use of existing EHR data, which had inherent limitations in terms of data accuracy and completeness.^13^, ^14^, ^15^ For example, CKD aetiology could not be accurately ascertained from the current dataset. Future research could consider a more detailed and nuanced analysis of CKD progression, including CKD progression in terms of declines in eGFR over time.^11^, ^16^ Using data from across all CKD stages over a longer follow‐up period would facilitate a deeper understanding of how age, gender, comorbidities and access to evidence‐based prevention influence kidney health outcomes.

## Supporting information


**Appendix S1.** Supporting Information.

## Data Availability

The data used in this study are subject to approval by individual data custodians (e.g. NT Health, individual ACCHOs). Access may be granted upon reasonable request and approval from the custodians, subject to ethical approval.
