# Supplementary material for: Rapid chronic kidney disease progression in younger, First Nations patients in the Northern Territory
Source: Intern Med J. 2025 Apr 22;55(5):860–3. doi: 10.1111/imj.70062 (PMC12077581; doi:10.1111/imj.70062)
Supplement: Supplementary file 1 — Appendix S1. Supporting Information. [file IMJ-55-860-s001.pdf]

Supporting information

Title: Rapid chronic kidney disease progression in younger, First Nations patients in the Northern Territory

Authors: Winnie Chen, Oyelola Adegboye, Gillian Gorham, Asanga Abeyaratne, Sandawana William Majoni, Sean Taylor, Samuel Heard, Harshana Munasinghe, Matthew J. L. Hare, Alan Cass

Contents:

I. Additional figures

- Figure S1: Kaplan-Meier survival curves for time-to KRT, by age category
- Figure S2: Kaplan-Meier survival curves for time-to death, by age category

II. Additional tables

- Table S1: Characteristics of included patients at baseline 2017, by age category
- Table S2: Proportion of individuals with KRT and death at the end of 6 years follow-up, by age category
- Table S3: Cox proportional hazard model results, for combined outcome of KRT or death
- Table S4: Cox proportional hazard model results, for KRT as an outcome
- Table S5: Cox proportional hazard model results, for death as an outcome
- Table S6: Table of cumulative incidence for competing risks of KRT and death

III. Territory Kidney Care steering committee

I. Additional figures

Figure S1: Kaplan-Meier survival curves for time-to KRT, by age category

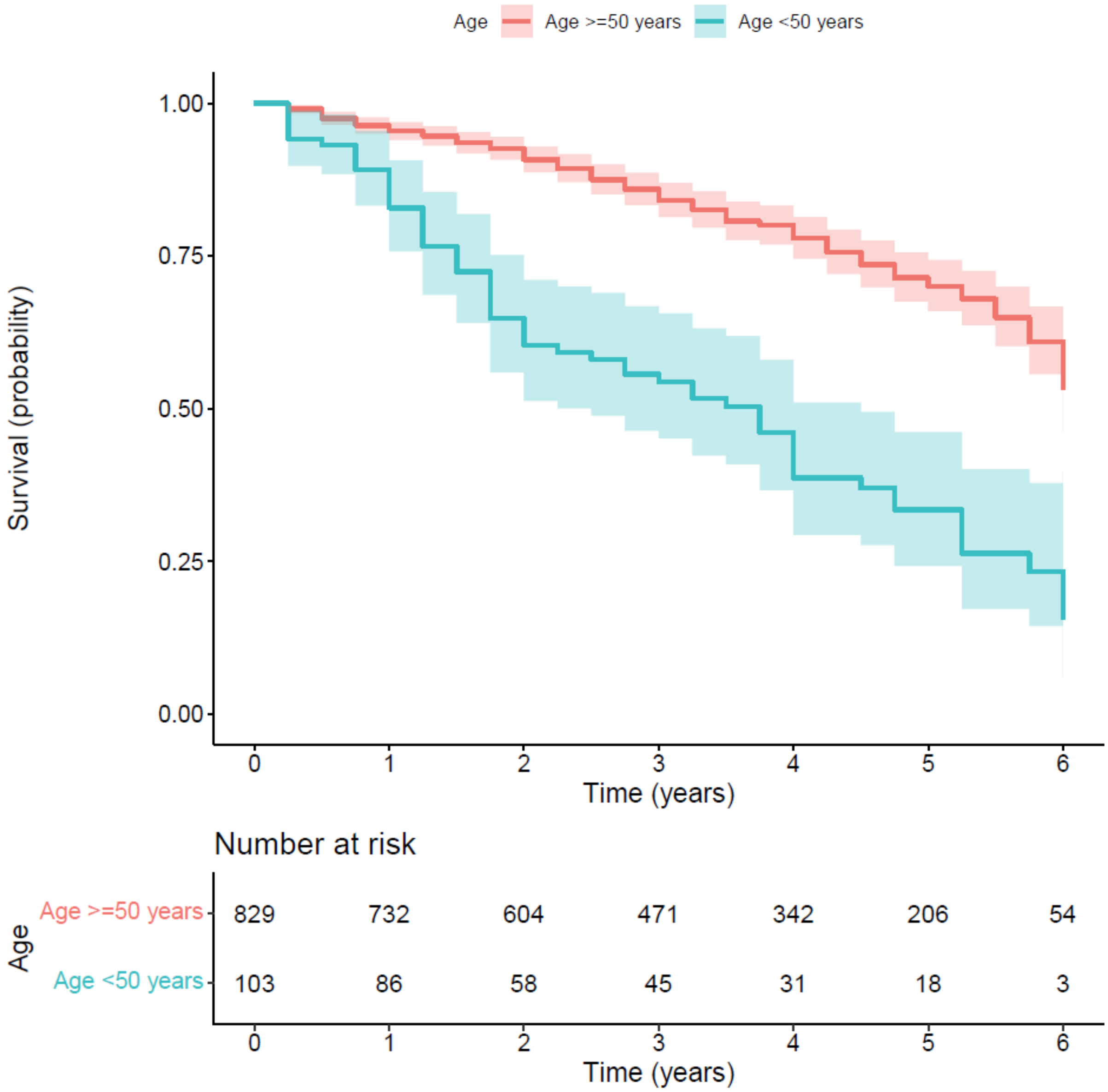

Red represents individuals age >= 50 years and blue represents individuals <50 years. The number at risk at each time interval, in years, is shown below the graphs.

Figure S2: Kaplan-Meier survival curves for time-to death, by age category

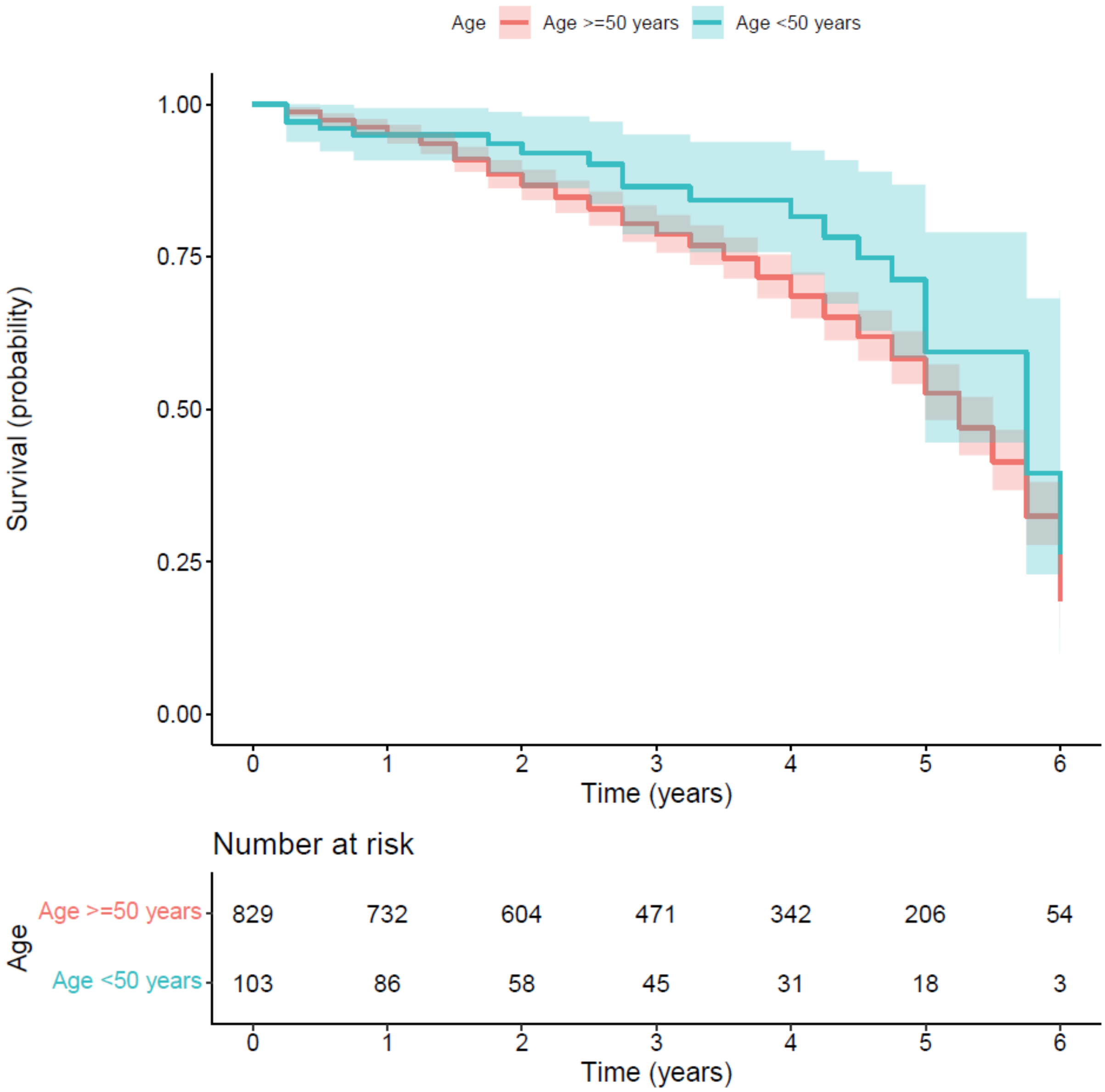

Red represents individuals age >= 50 years and blue represents individuals <50 years. The number at risk at each time interval, in years, is shown below the graphs.

II. Additional tables

Table S1: Characteristics of included patients at baseline 2017, by age category

Characteristic	Younger (<50 years) N = 130	Older ≥50 years) N = 999	All N = 1,129
Age	38 (9)	62 (17)	60 (19)
Gender (M)	55 (42%)	349 (35%)	404 (36%)
Baseline CKD stage			
• CKD 3A	65 (50%)	529 (53%)	594 (53%)
• CKD 3B	32 (25%)	250 (25%)	282 (25%)
• CKD 4	22 (17%)	146 (15%)	168 (15%)
• CKD 5	11 (8.5%)	74 (7.4%)	85 (7.5%)
Baseline eGFR*	43 (28)	44 (25)	44 (25)
Baseline uACR*	162 (333)	58 (207)	69 (238)
Comorbidities			
• Diabetes	78 (60%)	808 (81%)	886 (78%)
• Hypertension	96 (74%)	900 (90%)	996 (88%)
• Coronary artery disease	29 (22%)	386 (39%)	415 (37%)
• Cerebral vascular disease	5 (3.8%)	100 (10%)	105 (9.3%)
• Peripheral vascular disease	3 (2.3%)	49 (4.9%)	52 (4.6%)
• Obesity	43 (33%)	221 (22%)	264 (23%)

• Rheumatic heart disease	23 (18%)	134 (13%)	157 (14%)

*Missing values are excluded for eGFR (n=25 individuals) and uACR (n=186 individuals).
Median (IQR) for continuous variables; n (%) for categorical variables. Percentages rounded to 2 significant figures.
Abbreviations: CKD – chronic kidney disease; eGFR – estimated glomerular filtration rate; uACR – urine albumin creatinine ratio.

Table S2: Proportion of individuals with KRT and death at the end of 6 years follow-up, by age category

	**Younger (<50 years)** N = 130	**Older ≥50 years)** N = 999	**P-value**
Follow-up time (years)	3.8 (1.8 to 6.0)	5.8 (2.5 to 6.0)	<0.001
Status at 6 years*			<0.001
• CKD without KRT	46 (35%)	473 (47%)	
• KRT	72 (55%)	254 (25%)	
• Death before KRT	12 (9.2%)	272 (27%)	

*At the end of the follow-up period of 6 years.
**Wilcoxon rank sum test for continuous various and Pearson’s Chi-squared test for categorical variables.
Median (IQR) for continuous variables; n (%) for categorical variables. Percentages rounded to 2 significant figures.
Abbreviations: KRT – kidney replacement therapy.

Table S3: Cox proportional hazard model results, for combined outcome of KRT or death

Characteristic	Adjusted HR	95% CI (lower, upper)	p-value
Age category			
Younger (<50 years)	2.17	1.71, 2.75	<0.001
Older (≥50 years)	—	—	
Gender (M)	0.89	0.75, 1.05	0.162
Diabetes	1.72	1.37, 2.18	<0.001
Cardiovascular disease	1.2	1.02, 1.42	0.029
Hypertension	2.91	2.02, 4.21	<0.001

*Cardiovascular disease refers to any of the following: coronary artery disease, cerebral vascular disease, peripheral vascular disease. Abbreviations: HR – hazard ratio; CI – confidence interval.

Table S4: Cox proportional hazard model results, for KRT as an outcome

**Characteristic**	**Adjusted HR**	**95% CI (lower, upper)**	**p-value**
Age category			
Younger (<50 years)	4.92	3.66, 6.62	<0.001
Older (≥50 years)	—	—	
Gender (M)	0.98	0.75, 1.27	0.861
Diabetes	3.19	2.07, 4.91	<0.001
Cardiovascular disease	0.77	0.59, 1.00	0.051
Hypertension	9.36	3.82, 22.93	<0.001

*Cardiovascular disease refers to any of the following: coronary artery disease, cerebral vascular disease, peripheral vascular disease. Abbreviations: HR – hazard ratio; CI – confidence interval.

Table S5: Cox proportional hazard model results, for death as an outcome

Characteristic	Adjusted HR	95% CI (lower, upper)	p-value
Age category			
Younger (<50)	0.8	0.52, 1.24	0.323
Older (≥50)	—	—	
Gender (M)	0.82	0.65, 1.02	0.076
Diabetes	1.22	0.92, 1.62	0.174
Cardiovascular disease*	1.7	1.37, 2.11	<0.001
Hypertension	1.61	1.08, 2.41	0.02

*Cardiovascular disease refers to any of the following: coronary artery disease, cerebral vascular disease, peripheral vascular disease. Abbreviations: HR – hazard ratio; CI – confidence interval.

Table S6: Table of cumulative incidence for competing risks of KRT and death

Competing outcome	Year 1	Year 2	Year 3	Year 4	Year 5	Year 6	P-value
KRT							
Age category							<0.001
• Younger (<50 years)	13% (8.0%, 19%)	32% (24%, 40%)	37% (29%, 45%)	48% (39%, 56%)	52% (43%, 60%)	55% (46%, 63%)	
• Older	6.0% (4.6%, 7.6%)	11% (9.3%, 13%)	17% (14%, 19%)	20% (18%, 23%)	23% (20%, 26%)	25% (23%, 28%)	
Death							
Age category							<0.001
• Younger	3.8% (1.4%, 8.2%)	5.4% (2.4%, 10%)	7.7% (3.9%, 13%)	7.7% (3.9%, 13%)	9.2% (5.0%, 15%)	9.2% (5.0%, 15%)	
• Older	3.9% (2.8%, 5.2%)	9.2% (7.5%, 11%)	13% (11%, 16%)	17% (15%, 20%)	22% (20%, 25%)	27% (25%, 30%)	

*Fine-Gray test. N (%) for categorical variables, with 95% confidence intervals. Percentages rounded to 2 significant figures.
Abbreviations: KRT – kidney replacement therapy.

III. Territory Kidney Care steering committee

List of Territory Kidney Care steering committee members, in addition to authors.

1. Ali Lloyd – NT Primary Health Network

2. Andrew Bell – NT Health

3. Christine Connors – NT Health

4. Craig Castillon – NT Health

5. David McGuiness – Katherine West Health Board Aboriginal Corporation

6. Emma Kennedy – Pandanus Medical

7. Jenny Jobst – Miwatj Health Aboriginal Corporation

8. Liz Moore – Aboriginal Medical Services Alliance Northern Territory

9. Louise Maple-Brown – NT Health

10. Molly Shorthouse – NT Health

11. Nadarajah Kangaharan – NT Health

12. Nathan Garrawurra – Miwatj Health Aboriginal Corporation

13. Nathan Rosas – Wurli Wurlinjang Health Service

14. Pratish George – NT Health

15. Rama Nair – NT Cardiac Pty Ltd

16. Rebecca Bond – Sunrise Health Service Aboriginal Corporation

17. Robert Forbes – NT Health

18. Ronald Ogilvie – Sunrise Health Service

19. Satpinder Daroch – NT Department of Corporate and Digital Development

20. Velma King – Wurli Wurlinjang Health Service
